# Silver and Gold Nanoparticles Exposure to *In Vitro* Cultured Retina – Studies on Nanoparticle Internalization, Apoptosis, Oxidative Stress, Glial- and Microglial Activity

**DOI:** 10.1371/journal.pone.0105359

**Published:** 2014-08-21

**Authors:** Erika Söderstjerna, Patrik Bauer, Tommy Cedervall, Hodan Abdshill, Fredrik Johansson, Ulrica Englund Johansson

**Affiliations:** 1 Inst. Clinical Sciences, Dept. Ophthalmology, Lund University, Lund, Sweden; 2 Dept. of Biology, Sec. Functional Zoology, Lund University, Lund, Sweden; 3 Biochemistry and Structural Biology, Lund University, Lund, Sweden; Argonne National Laboratory, United States of America

## Abstract

The complex network of neuronal cells in the retina makes it a potential target of neuronal toxicity – a risk factor for visual loss. With growing use of nanoparticles (NPs) in commercial and medical applications, including ophthalmology, there is a need for reliable models for early prediction of NP toxicity in the eye and retina. Metal NPs, such as gold and silver, gain much of attention in the ophthalmology community due to their potential to cross the barriers of the eye. Here, NP uptake and signs of toxicity were investigated after exposure to 20 and 80 nm Ag- and AuNPs, using an *in vitro* tissue culture model of the mouse retina. The model offers long-term preservation of retinal cell types, numbers and morphology and is a controlled system for delivery of NPs, using serum-free defined culture medium. AgNO_3_-treatment was used as control for toxicity caused by silver ions. These end-points were studied; gross morphological organization, glial activity, microglial activity, level of apoptosis and oxidative stress, which are all well described as signs of insult to neural tissue. TEM analysis demonstrated cellular- and nuclear uptake of all NP types in all neuronal layers of the retina. Htx-eosin staining showed morphological disruption of the normal complex layered retinal structure, vacuole formation and pyknotic cells after exposure to all Ag- and AuNPs. Significantly higher numbers of apoptotic cells as well as an increased number of oxidative stressed cells demonstrated NP-related neuronal toxicity. NPs also caused increased glial staining and microglial cell activation, typical hallmarks of neural tissue insult. This study demonstrates that low concentrations of 20 and 80 nm sized Ag- and AuNPs have adverse effects on the retina, using an organotypic retina culture model. Our results motivate careful assessment of candidate NP, metallic or-non-metallic, to be used in neural systems for therapeutic approaches.

## Introduction

Retinal dysfunction caused by disease, damage or external factors often lead to visual loss. The retina is a complex organized structure at the back of the eye, including three nuclear neuronal layers and two synaptic layers. Sensory neurons, *i.e.* the photoreceptors, convert light to an electric signal that is transmitted through the bipolar cells and to the retinal ganglion cells, which axons form the optic nerve (ON) that send information from the retina to the brain for visual processing [1]. The neurons participating in this process are very vulnerable to deprivation of oxygen and nutrients, damage or foreign factors [2], [3].

In ocular research, enormous resources within the pharmaceutical industry and academia are allocated to investigate the use of material on the nano-scale for therapeutic applications, where *e.g.* nanoparticles (NPs) could serve as the active component or as a carrier for a functional agent [4]–[6]. NPs, especially small-sized (∼20 nm) render an enormous interest by having the ability to overcome the barriers of the eye, including the cornea, conjunctiva and blood-retinal barriers [4], [5], [7], [8]. Up-to-date several nano-sized materials have been applied in ocular research, spanning from metals, carbon, polymers and silica to materials of biological origin, such as lipids or lactic acid [7]. Both for commercial and clinical use, Au- and AgNPs, have been intensively studied, AuNPs due to their good intrinsic properties, *i.e.* high chemical stability, well-controlled size and surface functionalization, and AgNPs due to their antibacterial effect, often applied in wound disinfection, coatings of medical devices and prosthesis, and commercially in textiles, cosmetics and household goods [9].

The major eye diseases, age-related macular degeneration (AMD) and diabetes-related retinopathy involve abnormal formation of new blood vessels, leading to visual loss if untreated. Notably, Au- and AgNPs inhibit retinal neovascularization in experimental models, both as carriers of functional agents and in their native form [7], [10].

However, the literature on evaluation of adverse effects on ocular tissue, after Au- and AgNP exposure is sparse considering their widely use, especially within clinical care and diagnostics. In addition, since research on the therapeutic applications for various ocular diseases including AMD and diabetes-related retinopathy, are progressing fast there is an urgent need for the validation of unwanted side effect of candidate NPs using relevant models of the eye and the retina [4], [6], [11], [12].

*Ex vivo* and *in vitro* cultures of various organs, including neural tissue from the brain and the eye, have proven useful as intermediate complexity test systems in order to assess the potential use of candidate substances for therapy as well as for early signs of intolerable side-effects [13]–[15]. *In vitro* cultured retina can maintain its unique cell organization up to four weeks in culture and offers a controlled environment for initial testing of novel therapies [16].

Here we speculated whether 20 and 80 nm Au- and AgNPs in low concentrations are taken up by retinal tissue and cause adverse effects in *ex vivo* cultured post-natal mouse retina. After 72 h of NP exposure the retinas were examined for NP internalization using TEM, gross morphological abnormalities using routine histological staining, level of glial- and microglial activity, using glial specific markers and immunohistochemistry, effects on oxidative stress and apoptosis using biochemical assays.

## Methods

### Animals

Animals were kept under conditions with standard white cycling lightning, free access to food and water, and were used irrespectively of gender. C3H wild-type (wt) mice were used for the study. Mouse retinal tissues were taken from postnatal day 7 (PN7) animals. Animal handling was performed in accordance with approved guidelines of the Ethics Committee of Lund University, the Institute for Laboratory Animal Research (Guide for the Care and Use of Laboratory Animals), and the ARVO statement for the use of animals in ophthalmic and vision research.

### Nanoparticles and silver nitrate

Commercially available citrate stabilized colloidal gold and silver nanoparticles (AuNPs and AgNPs) of both 20 and 80 nm in diameter, respectively, in water were purchased from BBI International (Cardiff, UK). The particle sizes were measured from transmission electron microscopy images and presented as mean +/− standard deviation (n = 50). The AuNPs, diluted 10 times in ultrapure water, and AgNPs were characterized in water. The particle size was determined in triplicates by Dynamic Light Scattering (DLS) using a Dynapro Plate Reader II (Wyatt Technology, USA) and in duplicates by Differential Centrifugal Sedimentation (DCS) in a 24% to 8% sucrose gradient using a DC-24000 Disc Centrifuge (CPS Instrument Inc., USA). The reported hydrodynamic diameter by DLS is from cumulants analysis and the reported diameter from DCS is the peak value from the absorbance size distribution. The absorption spectra for the particles were recorded using a UV-800 spectrophotometer (Shimadzu, Japan). Prior to dilutions of the NPs the NP stock solutions were vortexed. For the experiments in tissue culture the dilutions were made in fresh R16 medium. Silver nitrate (AgNO_3_) (VWR International, Radnor, PA, USA) was dissolved in deionized water to give a stock solution of 1 mg/ml. The stock solution was sterile-filtered and stored at 4°C until use. Further dilutions were made in fresh R16 medium.

### *In vitro* retinal tissue culturing

Animals were sacrificed by an overdose of CO_2_. The eyes were enucleated; thereafter the anterior segment, the vitreous body and the sclera were removed. The neural retina with pigmented epithelium was explanted onto a Millicell-PCF 0.4 µm culture plate inserts (Millipore) with the vitreal side oriented upwards. The retinal explants were cultured in serum-free conditions in R16 culture medium (Invitrogen, Paisley, Scotland). Explants were allowed to adjust to culture conditions for two days *in vitro*, before receiving fresh R16 medium and addition of AuNPs, AgNPs, or AgNO_3_, and were then cultured for another 72 h. Au- and AgNPs, respectively, of either 20 nm or 80 nm were added to the medium to give the final concentrations; 0.0065 µg/ml 20 nm AuNPs, 0.4 µg/ml 80 nm AuNPs, 0.0035 µg/ml 20 nm AgNPs and 0.22 µg/ml 80 nm AgNPs. The concentrations chosen were based on our previous studies demonstrating uptake of the NPs in a human neural cell line, with the respective concentrations given corresponding to about 800 NPs/cell [17]. AgNO_3_ was added to the medium to give final concentrations of 0.5, 1.0 and 5.0 µg/ml.

Four independent culture experiments were performed on different days, rendering in total n = 5–8 explants/group.

### Tissue handling

For histology staining’s, retinas were fixated in 4% para-formaldehyde and then embedded in a gelatin medium (30% egg albumin and 3% gelatin in distilled water). Sections of 12 µm were cut with a cryostat, and stored at −20°C until further processing.

For transmission electron microscopy (TEM) analysis, the retinas were fixed in 2.5% glutaraldehyde in 0.15 M Na-cacodylate buffer (pH 7.2) for 4 hour at 4°C. After rinsing in 0.1 M Na-cacodylate buffer and dehydration, the retinas were post-fixed in 1% osmiumtetraoxide in 0.1 M Na-cacodylate buffer at 4°C for 1 hour. After dehydration, the samples were embedded in Epon. An ultramicrotome (Leica ultracut, Leica Microsystems GmbH, Germany) was used to cut ultrathin sections. The sections were stained with 2% uranyl acetate in PB-citrate [18].

### Histological staining

#### Hematoxylin-eosin staining

For gross morphological analysis every tenth section throughout all specimens was stained with Hematoxylin-eosin (Htx-eosin). Sections were coverslipped, using Pertex mounting media (HistoLab, Sweden).

#### Fluorescent immunostaining- glial- and microglial activity

For immunostaining six-eight sections per specimen (together representing the whole specimen) were rinsed and then pre-incubated incubated in phosphate buffered saline containing 0.1% Triton X-100 (PBST), 1% bovine serum albumin (BSA), and 5% normal donkey serum (Jackson ImmunoResearch Laboratories Inc., West Grove, PA, USA) for 1 h at room temperature. Sections were then incubated with primary antibodies, rabbit anti-glial fibrillary acidic protein (GFAP, 1∶1500 DAKO Cytomation, Glostrup Denmark), rabbit anti- Iba1 (1∶200, WAKO, Japan), and rat anti-mouse ED1 (CD68, 1∶1000, Nordic Biosite, Sweden), 1∶1000, overnight at 4°C overnight, and thereafter detected by incubation in secondary antibodies for 2 h. Secondary antibodies included were Texas Red-conjugated donkey anti-rabbit antibody (1∶200; Abcam, Cambridge, UK), Alexa 488 goat anti-rabbit IgG (Molecular Probes) and Alexa 564 goat anti-rat (Molecular Probes). Both primary and secondary antibodies were diluted in PBST containing 1% BSA. For counterstaining of nuclei, the sections were cover-slipped using 4′6- diamidino- 2- phenylindole (DAPI)-containing Vectashield mounting medium (Vector Laboratories, Burlingame, CA, USA).

#### TUNEL assay- Staining for apoptotic cells

Eight sections per specimen (together representing the whole specimen) were stained with a fluorescein-conjugated terminal deoxynucleotidyl transferase-mediated dUTP nick end-labeling (TUNEL) assay according to the manufacturer (Roche, Mannheim, Germany). For counterstaining of nuclei, the sections were cover-slipped using DAPI-containing Vectashield mounting medium.

#### AvidinD staining- staining for oxidative stressed cells

Six sections per specimen (together representing the whole specimen) were stained with AvidinD (1∶400, Texas Red–conjugated avidin; Molecular Probes Inc., Eugene, OR, USA; A-820). AvidinD was diluted in PBST containing 1% BSA and sections were incubated for 45 min at RT. For counterstaining of nuclei, the sections were cover-slipped using DAPI-containing Vectashield mounting medium.

### Overall analysis, quantifications and data analysis

#### TEM analysis

For TEM analysis, samples from one experiment were evaluated, and detailed analysis was performed throughout the entire retina (from inner to outer retina). A minimum of 50 cells per nuclear layer was in-depth analyzed for investigation of intracellular localization of the respective NP. Sections were imaged in a JEOL JEM 1230 microscope (JEOL, Japan).

#### Morphological analysis

Gross- as well as detailed morphological analysis of every tenth section throughout the retinal explants (n = 5–8/group) was performed using light-and fluorescent microscopy (Nikon, Tokyo, Japan). The following parameters were included in the evaluations; retinal layering, fold formation, rosette formation, specific changes in nuclear layer morphology and presence of pyknotic cells. Counter-stained and immunostained sections of the retinal explants were examined using an epifluorescence microscope (Nikon Eclipse E800, Japan) equipped with appropriate filters. Images were captured with digital acquisition system (DCP Controller).

#### Glial reaction

Analysis of changes of GFAP expression and staining pattern was performed (n = 5–8/group and eight sections per specimen). In addition, gross- and detailed structural morphologies of the GFAP-labeled cells were performed. No quantitative analysis was made.

#### Microglial activation

Microglial cells were double immune-labeled using the microglial-specific markers Iba1 and ED1 (n = 4/group and six sections per specimen). Total numbers of cells were quantified throughout the entire retina without subdividing it into specific nuclear layers.

At first, the total numbers of microglial cells per specimen were quantified by counting the total numbers of microglial cells, detected by the expression of Iba1/ED1 or ED1.

Secondly, the numbers of Iba1-positive cells (expressed in all stages of microglia activation) expressing the marker for activated microglial cells, *i.e.* ED1, were quantified.

Thereafter, the morphological change occurring in response to activation of microglia was used to assess the level of activation. The following morphological classification for activation stage was used:

*Ramified* (round cell body, long branched processes)* = *resting stage.

*Intermediate* (elongated cell body, with short thick non branched processes) = mid-activated stage.

*Round* (round cell body, no processes) = active stage.

*Amoeboid* (irregular/ellipsoid cell body, no process) = active stage.

#### Numbers of apoptotic cells

Quantifications of total numbers of cells expressing the marker for apoptotic cells, TUNEL, were made (n = 5–8/group and eight sections per specimen). Quantifications of cells were made per nuclear layer, *i.e.* the ganglion cell layer (GCL), inner cell layer (INL) and outer nuclear layer (ONL).

#### Numbers of oxidative stressed cells

Initial analysis of the AvidinD staining revealed only positive stained cells in the ONL. Hence, quantification of AvidinD-positive cells was limited to this region of the retina (n 4/group and six sections per specimen).

#### Statistical analysis

Quantifications were performed using Image J64 and all data was normalized to cells per mm^2^. The area was measured using the DAPI staining, when applied. All data is presented as mean ± SD, t-tests were performed and *p* values given as * *p*≤0.05 and ** *p*≤0.01. *p* values ≤0.05 were considered statistically significant.

## Results

### Particle characterization

**Figure 1:**
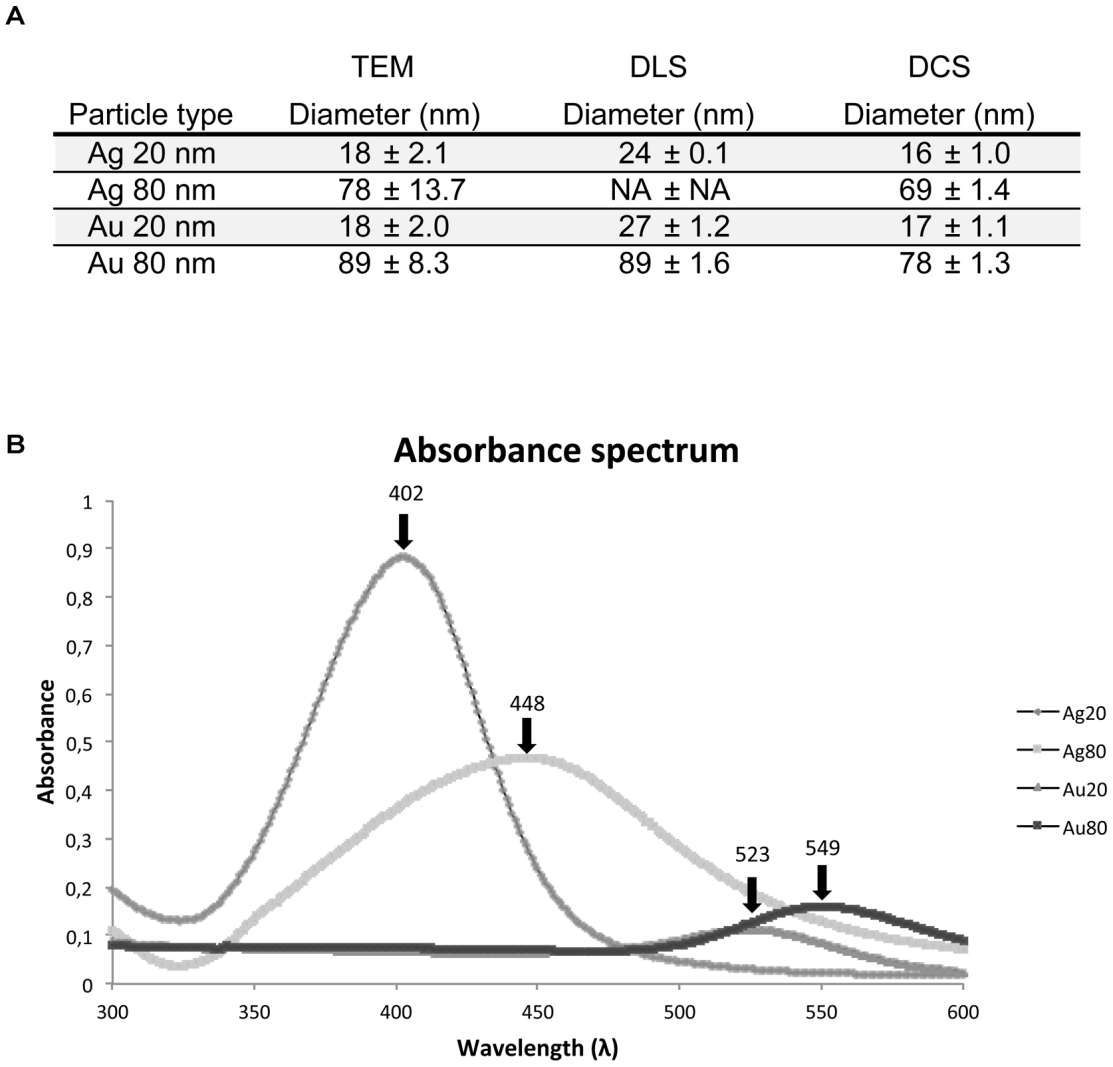
Nanoparticle characterization. A. The table shows the diameters of the commercially available NPs used in this study, measured by TEM, DLS and Differential Centrifugal Sedimentation. From TEM images the mean diameters of 50 particles ± standard deviation (SD) for each particle type are shown. The DLS data represent the hydrodynamic diameter from cumulants analysis of triplicate experiments and Differential Sedimentation diameters are the peak value from the absorbance size distribution of duplicate experiments with identical results. B. In the graph the absorption spectra of the respective NP is shown and the peak value is given for each NP.

**Figure 2:**
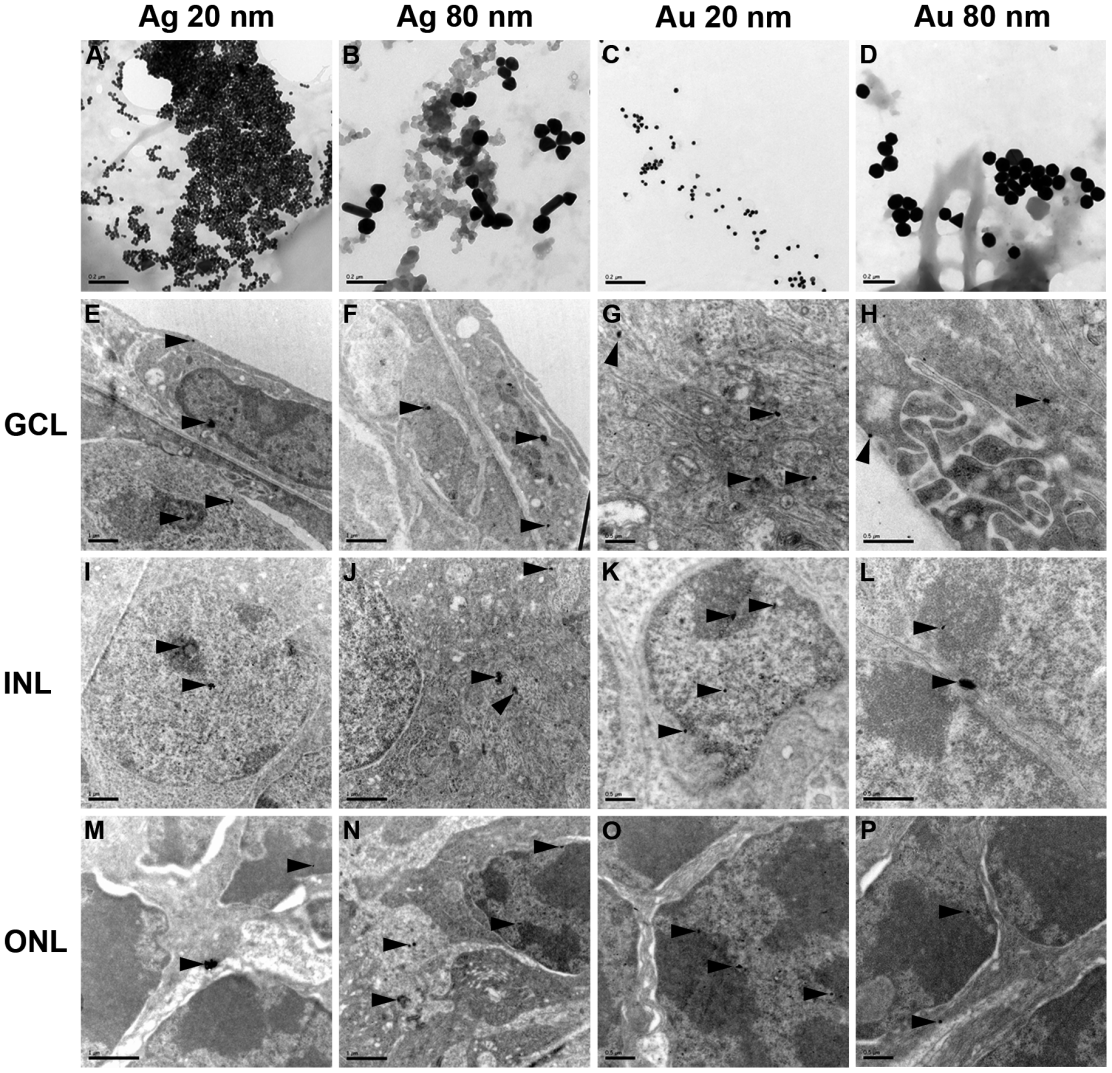
TEM-images demonstrating uptake of Ag- and AuNPs in the mouse retina. The top row shows the respective NP structure, *i.e.* AgNP 20 nm (A), AgNP 80 nm (B), AuNP 20 nm (C) and AuNP 80 nm (D). E–P. All four types of NPs were taken up by the cultured retina and found in all three retinal nuclear neuronal layers, *i.e.* the GCL, INL and ONL. Both 20 and 80 nm sized NPs were found either as single NPs (*e.g.* E, M (upper arrowheads) or as clusters of NPs (*e.g.* E, I, M (lower arrowheads). The 20 nm sized Ag- and AuNPs, respectively, were found in the nucleus (E, G, I, K, M, O), the nucleolus (I, K, O), the mitochondria (G), the cytosol (E, G, M) and in the extracellular space (M). In the majority of cells the largest fraction of the 20 nm sized NPs were located in the nucleus (I, K, O) compared to the fraction of NPs found in the other cell compartments. Notably, all NPs were found both in the eu- (*e.g.* I, K, N) and hetero-chromatin (*e.g.* I, K, L, P, M). Ag- and AuNPs, sized 80 nm, were detected in the nucleus, but to a lesser extent compared to the 20 nm NPs (F, J, L, N, P) and in the extracellular space (H, L, P). NPs, sized 80 nm, were not detected in the nucleolus and within mitochondria. GCL = ganglion cell layer, INL = the inner nuclear layer, ONL = the outer nuclear layer. Arrowheads show NPs that have been taken up by the retinal cells. Scale bars equal 0.2 µm for images A–D; 1 µm for E–F, I–J, and M–N; and 0.5 µm for G–H, K–L, and O–P.

We characterized the particles with regard to size and material. Our results were compared to those given by the purchaser (BBI, UK). The diameters of the respective particles were measured using four different methods, *i.e.* manually from TEM images, DLS, DCS, and by absorption spectra. The details of the particle sizes measured from the TEM images are found in Fig. 1A. The diameters of the four different NPs were measured from TEM images (Figs. 1A, 2A–D) and were indeed closely similar to the values 20 and 80 nm, provided by BBI, UK. The larger particles, both Au and Ag, were found to contain a portion of non-spherical particles (Figs. 2B and D). These non-spherical particles were included in the size measurements and influenced the standard deviations, which were found to be larger than for the smaller particles. The particle size was also determined in suspension by DLS and by DCS, Figure 1A. The sizes of the particles in suspension are similar to the sizes found by TEM. The Ag80 particles were too polydispersed to obtain DLS data. The polydispersity can be explained by the non-spherical particles observed by TEM. The maxima in the absorption spectra obtained here for the respective particle corresponded exactly for three of the NPs, *i.e.* Ag20 (402 nm), Au20 (523 nm) and Au80 (549 nm) and very close for Ag80 (448 nm (here) cf. to 451 (BBI, UK) (Fig. 1A). We conclude that the size and surface properties of the particles used here are in line with those reported by the manufacturer and with what is previously described for similar particles.

### Cellular and nuclear uptake of 20 and 80 Ag- and AuNPs

Due to their high atomic number, it is possible to distinguish Ag- and AuNPs from cellular structures using TEM [2], [3], [17]. In general, Ag- and AuNPs of both sizes were taken up by the retinal tissue and found distributed in all neuronal layers of the retina (the ganglion cell layer (GCL, Figs. 2E–H), the inner nuclear layer (INL, Figs. 2I–L), and outer nuclear layer (ONL, Figs. 2M–P)), judged from careful overall scanning through all the layers of the retina including detailed high magnification analysis of at least 50 cells/experimental group.

The 20 nm sized Ag- and AuNPs showed a similar internalization pattern, with NPs found in the nucleus (Figs. 2E, I, K, M, O), the nucleolus (Figs. 2I, K, O), the mitochondria (Fig. 2G) and in the cytosol (Figs. 2E, G, M) as well as in the extracellular space (Fig. 2M). The 20 nm sized NPs were found as single particles (*e.g.* Fig. 2K) and clusters of NPs (*e.g.* Fig. 2M). In some cells the 20 nm sized NPs were found in a larger fraction within the nucleus (Figs. 2I, K, O) compared to the fraction of NPs found in the other compartments of the cell.

The 80 nm Ag- and AuNPs were also detected in the nucleus, but overall to a lesser extent compared to their smaller-sized counterparts (Figs. 2F, J, L, N, P). The 80 nm sized NPs were also found as single particles (*e.g.* Figs. 2F, H, N, P) and clustered (*e.g.* Fig. 2L) NPs. However, 80 nm NPs were not detected in the nucleolus and within mitochondria, as was the case for the 20 nm sized NPs.

### Altered retinal gross morphology after 20 and 80 nm Ag- and AuNP exposure

Gross morphological analysis was performed on sections representing the entire retina, for signs of toxicity (n = 5–8 explants/group). The following parameters, which are typically affected in response to stress in the cultured retina, were included in the evaluations; retinal layering, fold formation, rosette formation, specific changes in nuclear layer morphology and presence of pyknotic cell nuclei. The presence of pyknotic nuclei is described qualitatively and compared to control. Nuclei with condensed chromatin are typically referred to as pyknotic, and thus likely the cell is undergoing apoptosis or necrosis [19], [20].

**Figure 3:**
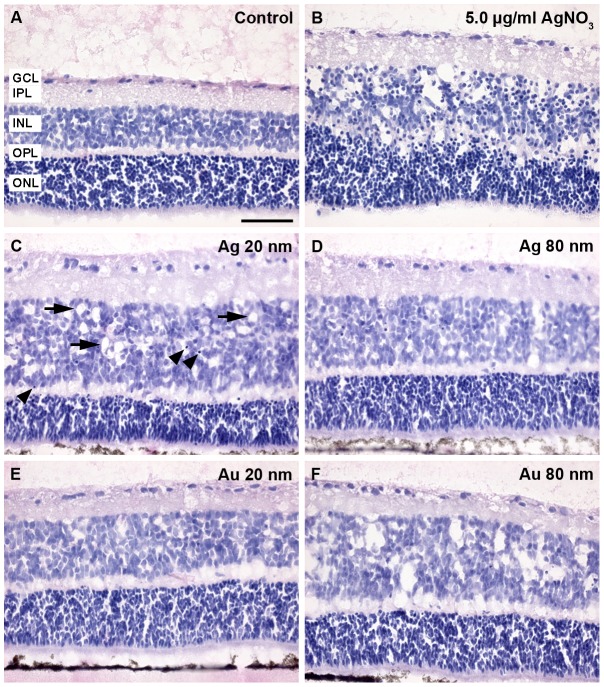
Gross morphological changes of the mouse retina after exposure to 20 and 80 nm Ag- and AuNPs. The photomicrographs show the representative gross morphology of control- and nanoparticle (NP) NP-exposed retinas, revealed by using Htx-Eosin staining (n = 5–8 retinas/group). A. The control retina displayed normal and distinct retinal layering. B. The positive control groups for toxicity by treatment with AgNO_3,_ *i.e.* 0.5, 1.0 and 5.0 µg/ml, caused severe insult to the retinal tissue and cells, with disrupted retinal layering, folding and rosette formation, formation of vacuoles, as well as an induction of massive cell death revealed by the large numbers of pycnotic cells found compared to the control. C–F. Exposure to 20 and 80 nm Ag or AuNPs caused no disruption of the retinal layering, fold- or rosette formation. However, after exposure to all four types of NPs large vacuoles in the tissue could be found, especially in the INL (arrows in C). A larger fraction of pycnotic cells especially in the INL were found for all treated groups, compared to control (arrowheads in C). GCL = ganglion cell layer, INL = inner nuclear layer, IPL = inner plexiform layer, ONL = outer nuclear layer, OPL = outer plexiform layer. Scale bars equal 50 µm.

All control retinas displayed normal retinal layering with the three nuclear layers intact, *i.e.* the GCL, INL and ONL as well as the synaptic layers, *i.e.* the inner- and outer plexiform layers (IPL, OPL), respectively, and no obvious sign of toxic insult to the overall tissue or cells (Fig. 3A) were found.

Positive control groups for toxicity caused by silver ions were included by treating retinas with three different concentration of AgNO_3_, *i.e.* 0.5, 1.0 and 5.0 µg/ml. All concentrations used caused very similar and severe insult to the retinal tissue and cells (Fig. 3B). With regard to the retinal layering, the synaptic layer OPL was no longer visible and the INL and ONL had in some regions merged into one layer. Moreover, AgNO_3_ exposure caused disruption of especially the ONL and INL, with large vacuoles found in the INL. In addition, retinal folding and rosette formation were frequently found. Massive cell death was induced after AgNO_3_ exposure as revealed by the large numbers of pyknotic cells found compared to the control.

Exposure to 20 and 80 nm Ag- or AuNPs caused no disruption of the retinal layering, fold- or rosette formation (Figs. 3C–F). However, after exposure to all tested NPs, large vacuoles in the tissue could be found, especially in the INL (Figs. 3C–F). No attempts were made to quantify the thickness of the NP-exposed retina for comparison to control, as the effect was obvious.

A larger fraction of pyknotic cells especially in the INL were found for all treated groups, compared to control. The dense and dark staining of the ONL made it difficult to judge the fraction of pyknotic cells in this layer for all groups (Figs. 3C–F). Ag- or AuNP exposure tended to make cells in the ONL more densely packed, and also more intensely stained compared to control, however, not pyknotic.

### Glial activation

Here GFAP-immunohistochemistry was used to label two types of glial cells in the retina, with the astrocytes mainly found within the nerve fiber layer (NFL) in the inner retina and the Müller glial cells with their cell bodies localized in the INL and their irregular fibers spanning the entire retina (n = 5–8 explants/group). The end feet of the Müller cells form the outer and inner limiting membrane, respectively, of the retina (OLM and ILM, respectively). In the normal retina GFAP-expression is typically detected in the Müller cell processes in the inner half of the retina and in their end feet. Increased glial activity, including morphological changes of the glial cells and massive up regulation of intermediate filament protein expression throughout the entire cell is well-described hallmarks of an insult to the retina [21], [22].

**Figure 4:**
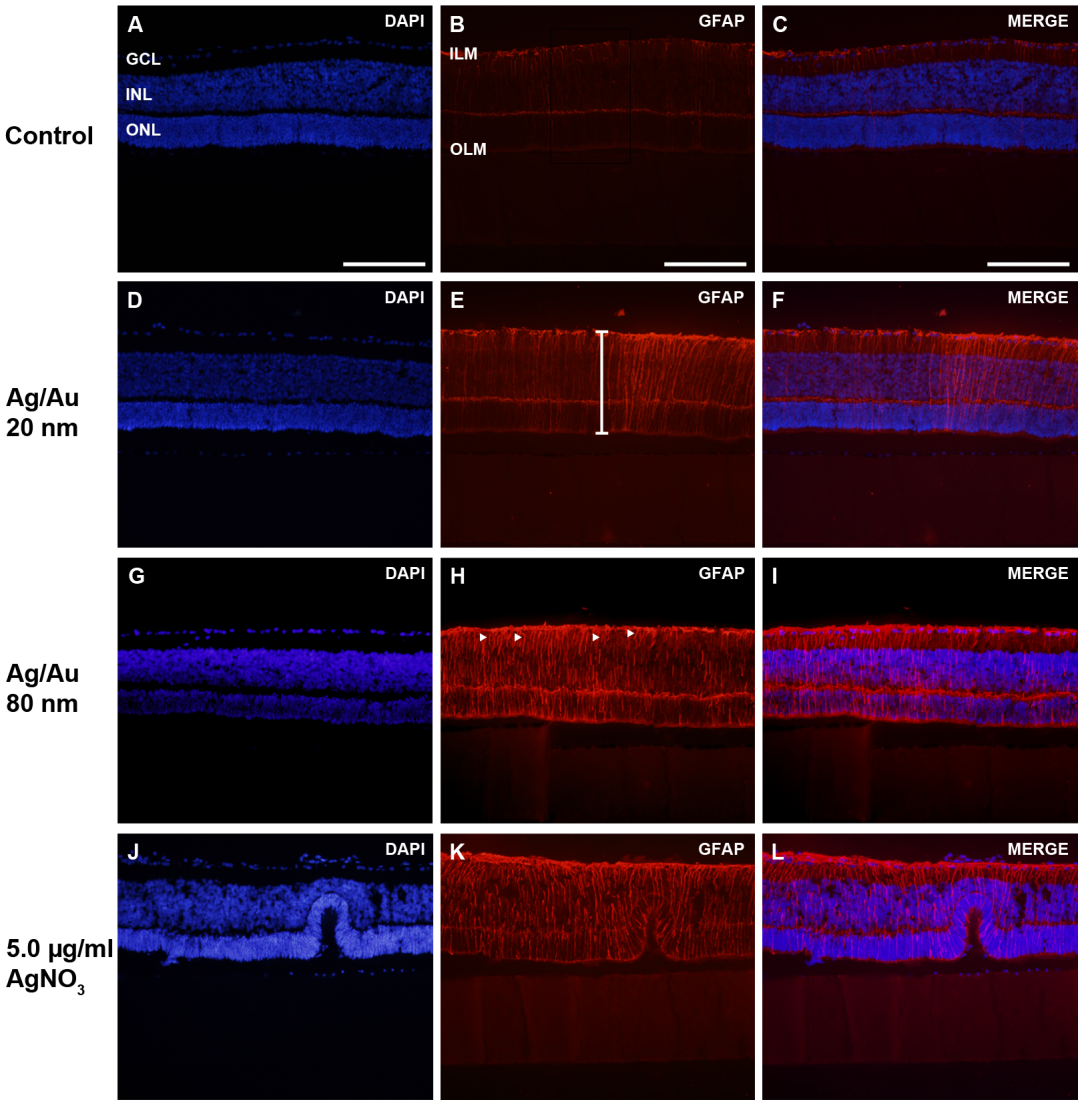
Increased GFAP-expression in the mouse retina after exposure to 20 and 80 nm Ag- and AuNPs. A–C. Control retinas displayed an even and low intensive GFAP-staining, with GFAP-expression detected in thin filaments in the inner half of the retina and in end feet in the ILM (n = 5–8 explants/group). Some GFAP-positive processes span the entire retina from the ILM to the OLM. D–F. Exposure to 20 nm sized Ag-or AuNPs caused increased expression of GFAP, seen by a larger fraction of GFAP-positive fibers as well as a mix of thin and thick fibers spanning the entire retina from the ILM to the OLM. G–I. Exposure to 80 nm NPs triggered glial activation even further than the 20 nm NPs, judged by the more intensive GFAP-staining and larger areas densely packed thick GFAP-expressing fibers from the OLM to the ILM. J–L. AgNO_3_-treated retinas, at all concentrations studied (0.5, 1.0 and 5.0 µg/ml) caused severe insult to the retinal tissue as shown by an intensive GFAP-staining of predominantly thick, short and irregular fibers as well as a densely stained ILM. GCL = ganglion cell layer, ILM = inner limiting membrane, INL = inner nuclear layer, OLM = outer limiting membrane, ONL = outer nuclear layer. Scale bar equals 200 µm.

All control retinas displayed a low and even GFAP-staining, with GFAP-expression primarily detected in glial filaments in the inner half of the retina as well as in the end feet in the ILM. Occasionally, GFAP-positive processes were found spanning the retina from the ILM to the OLM (Figs. 4A–C). The ILM and NFL were moderate stained in intensity and only scattered horizontal GFAP-positive cells were found (Fig. 4B).

Retinas exposed to 20 nm Ag- or AuNPs revealed very similar signs of glial activation with an overall up-regulation of GFAP expression, judged by a higher fraction of GFAP-positively stained fibers and the staining being more intense compared to the controls (Figs. 4D–F). In addition, thin fibers as within the controls, but also thick GFAP-processes were found after exposure to 20 nm NPs (Fig. 4E). Compared to control, larger areas of 20 nm Ag- or AuNP-treated retinas contained GFAP fibers spanning the entire retina including regions with a substantial number of thick fibers.

Exposure to 80 nm NPs triggered glial activation even further than the 20 nm NPs, judged by the more intensive GFAP-staining and larger areas densely packed with thick GFAP-expressing fibers displaying a short irregular shapes as well as spanning the retina from the OLM to the ILM. No difference in level of glial activation was noted between the groups exposed to 80 nm Ag- or AuNPs, respectively (Figs. 4G–I). In addition, the Müller cell end feet in both the ILM and OLM were very intense stained (Fig. 4H).

All AgNO_3_-treated retinas, at all concentrations studied (0.5, 1.0 and 5.0 µg/ml) caused severe insult to the retinal tissue as shown by an even and intensive GFAP-staining of predominantly thick, short and irregular fibers as well as a densely stained ILM (Fig. 4K).

### Microglial activation

In the healthy retina the microglial cells are distributed primarily in the IPL and OPL, where they are responsible for sensing pathological changes in their microenvironment [23]. Mertsch *et al* (2001) described the post-natal rodent retina culture model as very useful for studying microglial activity in response to an inflammatory stimulus [23].

Microglial activity in the retina in response to *e.g.* injury is associated with proliferation, migration, phagocytosis and release of many different bioactive molecules [24]. Activated microglia changes their morphology from ramified (resting) via an intermediate (mid-active) to amoeboid/round (active).

Here we investigated if exposure to the different types of NPs and AgNO_3_-treatment retinas induce microglial activity. The microglial-specific markers Iba1 [25] and ED1 ( = CD68) [26] were used. The Iba1 protein has a role in calcium homeostasis and is often expressed in all phases of the microglial activation, whereas ED1 is expressed in activated stages and is involved in phagocytosis.

**Figure 5:**
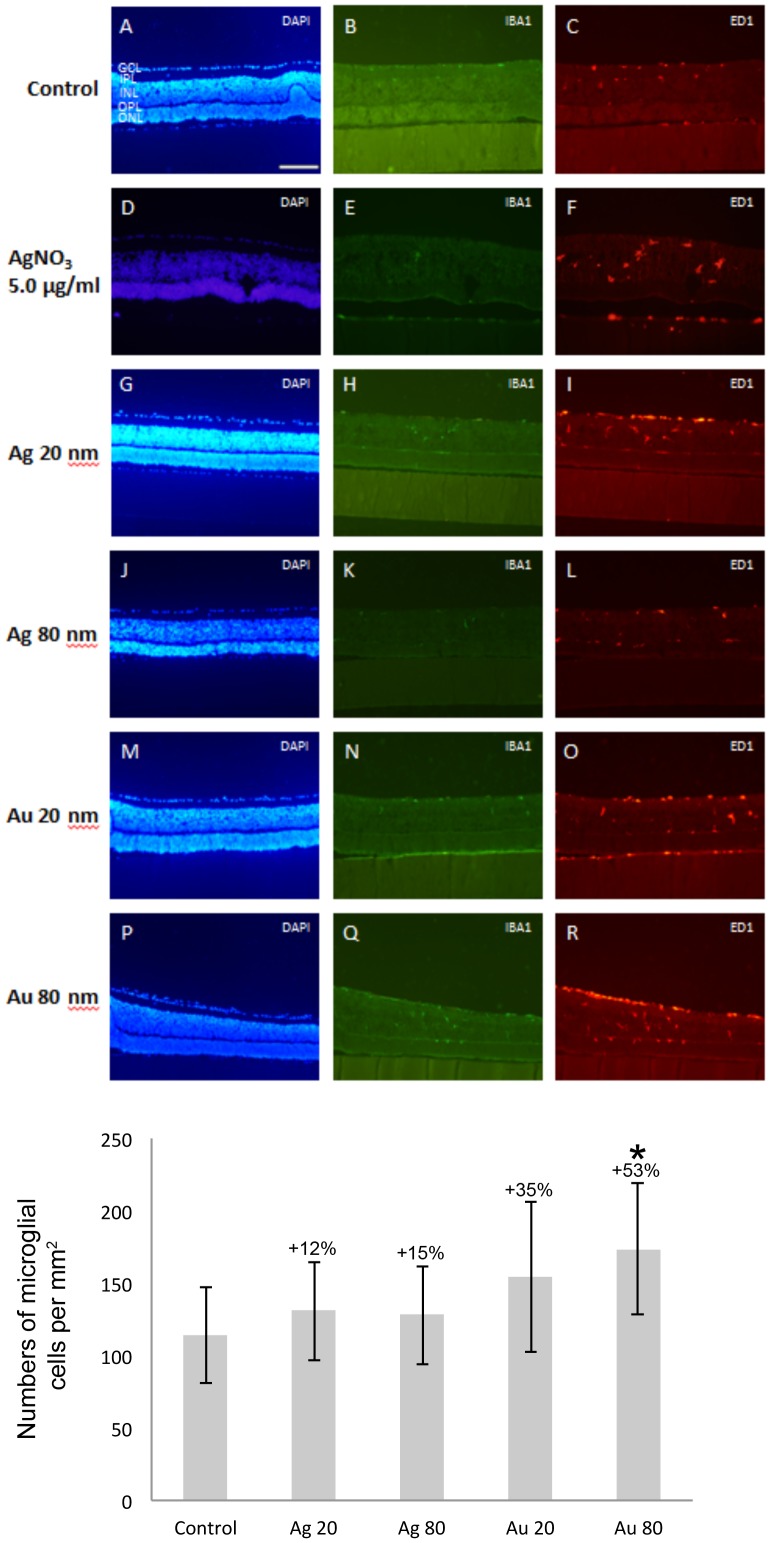
Increased proliferation of microglial cells in the mouse retina after exposure to 20 and 80 nm Ag- and AuNPs. Fluorescent immunostaining: Iba1 (green) and ED1 (red) for detecting microglial cells (DAPI, blue). In the NP-exposed retinas (20 nm Ag (G–I), 80 nm Ag (J–L), 20 nm Au (M–O), 80 nm Au (P–R)) as well as in the control retinas (A–C) the Iba1- and ED–positive cells were located in the IPL, INL and GCL in all groups (A–C). AgNO_3_-exposed retina displayed the same staining pattern, but with a stronger intensity especially for the ED1-staining (D–F). Graph shows numbers of microglial cells and data is given as mean ±SD (n = 4 explants/group). * *p*<0.05 compared to control. GCL = ganglion cell layer, INL = inner nuclear layer, IPL = inner plexiform layer, ONL = outer nuclear layer, OPL = outer plexiform layer. Scale bar equals 200 µm.

Overall, and in all groups Iba1- and ED1-positive cells were located within the IPL, INL and GCL (n = 4 explants/group, Figs. 5A–R). Exposure to all types of NPs increased the total numbers of microglial cells, *i.e.* Iba1+/ED1+- and Iba1−/ED1+ cell fractions (Fig. 5). AgNPs sized 20 nm and 80 nm gave a 15% and 12% increase, respectively. A 35% increase in cell number was found after exposure to 20 nm AuNPs, and a significant increase of 53% was noted after 80 nm AuNPs exposure. The total cells number per group is given as numbers of ED1-positive cells, since all Iba1-positive cells expressed of this marker.

Thus, our attempt to assess the microglial activity in the different groups based on the expression of ED1, a marker that is well described to only be found in activated cells, was not successful. All of the Iba1-positive cells were also found to express ED1, indicating that all microglial cells were at least in an early-activated stage.

**Figure 6:**
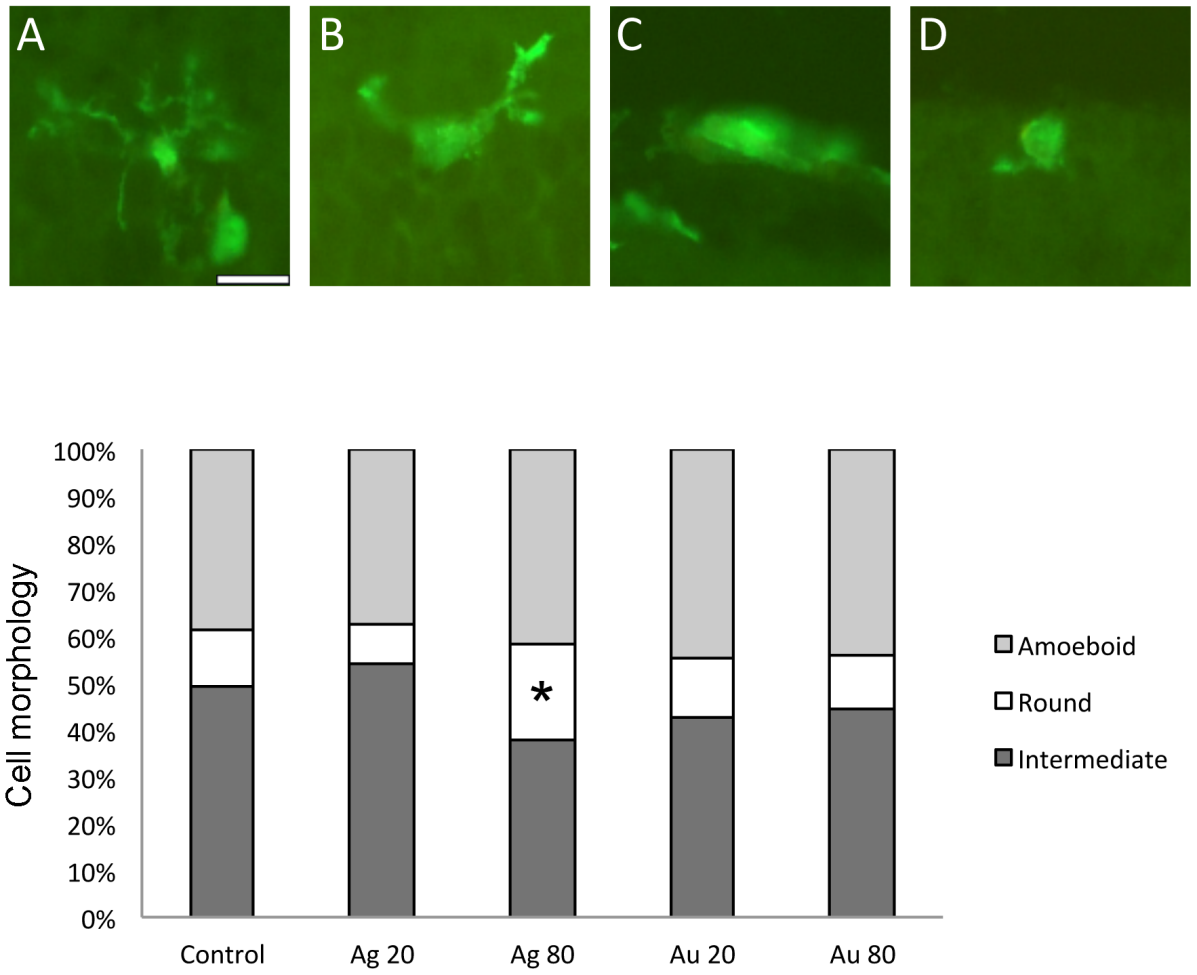
Morphological changes of microglia in the cultured mouse retina after exposure to 20 and 80 nm Ag- and AuNPs. A–D. Iba1-immunohistochemistry revealing microglial cells with ramified (A), intermediate (B), round (C) and amoeboid (D) morphology, respectively. Quantification of the fractions of microglia in three different stages of activation was performed: intermediate or “early activated” cells as judged from the morphology and co-expression of ED1 while round- and amoeboid morphologies are regarded as “late activated” cells. The graph shows the distribution of activation stages, normalized to total numbers of Iba1-positive cells/explant; values ±SD (n = 4 explants/group) are given. * *p*<0.05 compared to control. GCL = ganglion cell layer; INL = inner nuclear layer; ONL = outer nuclear layer. Scale bars equal 50 µm.

The morphological change occurring in response to activation of microglia was used to assess the level of activation. Iba1/ED1- or ED1-positive cells were classified into four different groups based on their morphology, *i.e.* ramified (Fig. 6A), intermediate (Fig. 6B), round (Fig. 6C) or amoeboid (Fig. 6D). However, the rarely found cells with a ramified profile always expressed the activated microglial marker ED1, and were therefore classified as intermediate in all experimental groups.

The fraction of cells exhibiting the intermediate morphology, here judged as the earliest activated cells, decreased in all NP exposed groups except for the group exposed to 20 nm AgNPs (Fig. 6E). Especially, the group exposed to 80 nm AgNPs, displayed a significant 30% increase in the fraction of activated microglia. Moreover, a 15% and 11% increase of activated cells were seen after exposure to 20 and 80 nm AuNPs, respectively.

### Ag- and AuNP exposure induce apoptosis

**Figure 7:**
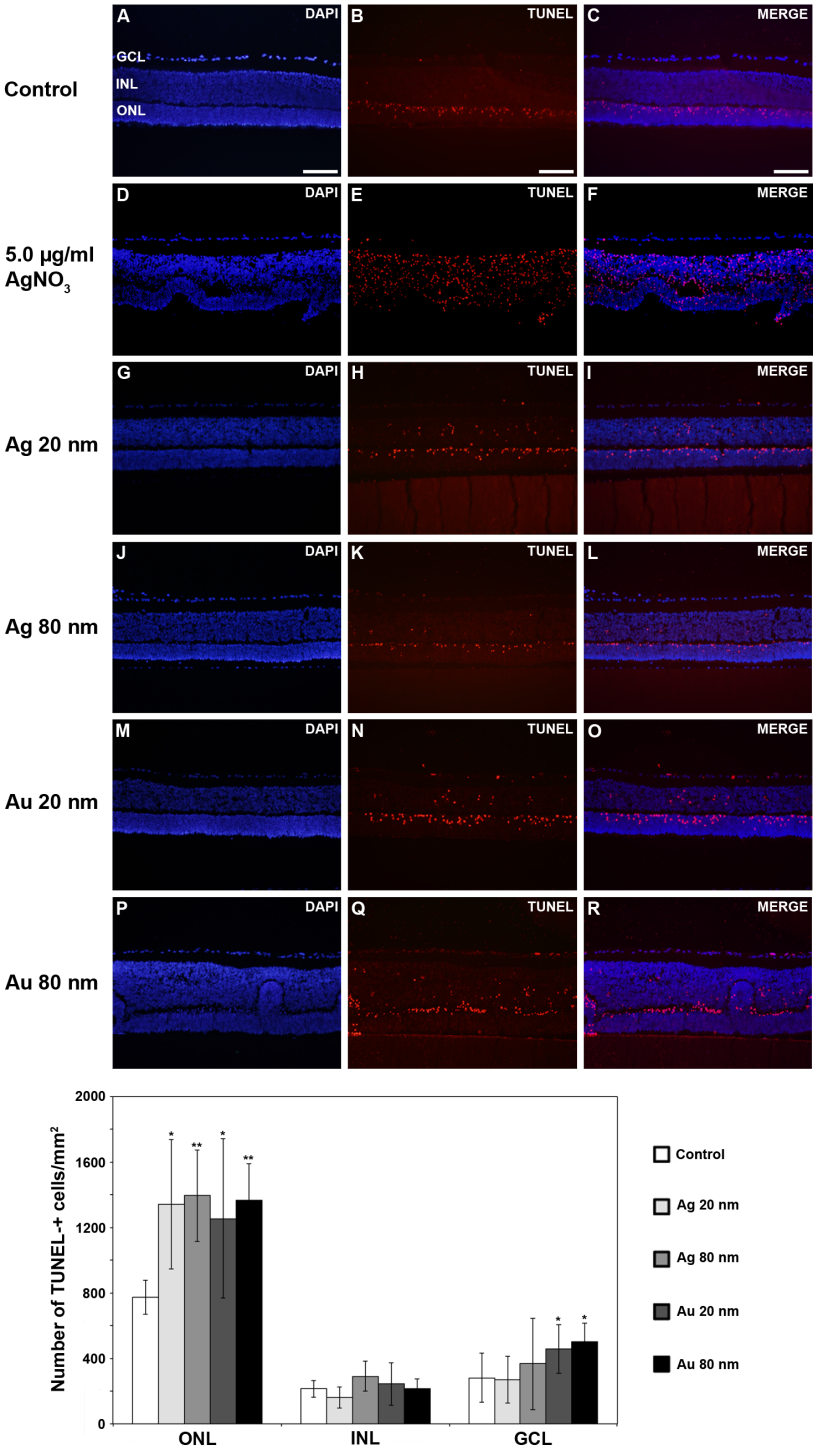
Increased number of apoptotic cells in the mouse retina after exposure to 20 and 80 nm Ag- and AuNPs. Apoptotic cells were detected using the biochemical assay TUNEL, and TUNEL-positive cells were detected in the ONL, INL and GCL in all group included, *i.e.* the controls (A–C), AgNO_3_-treated retinas (D–F), retinas exposed to 20 nm AgNPs (G–I), 80 nm AgNPs (J–L), 20 nm AuNPs (M–O) and 80 nm AuNPs (P–R), respectively. Graph shows numbers of TUNEL-positive cells and results are presented as mean ±SD (n = 5–8 explants/group). * *p*<0.05 compared to control, ** *p*<0.01 compared to control. GCL = ganglion cell layer, INL = inner nuclear layer, ONL = outer nuclear layer. Scale bars equal 100 µm.

By using a fluorescein-conjugated dUTP TUNEL-staining, we investigated the direct cytotoxic effects of Ag- and AuNPs on a cellular level. TUNEL-staining is commonly used to detect DNA fragmentation, which is a hallmark of apoptosis [17]. The numbers of apoptotic cells were quantified for each nuclear layer in the retina, *i.e.* the ONL, INL and GCL (n = 5–8 explants/group, see Figs. 7A–C for orientation). In the control retina some apoptosis was found in all layers, which is expected since the explantation process itself is causing a stress response in the tissue [16]. The massive number of TUNEL- positive cells confirmed AgNO_3_-toxicity by free Ag ions (Figs. 7D–F). Although, the numbers of apoptotic cells were not quantified for the AgNO_3_- treated retinas.

Overall, TUNEL-positive cells were detected in all groups, including control and treatment groups, with the highest numbers of TUNEL-positive cells found in the ONL followed by the GCL and the INL.

In the ONL, the number of TUNEL-positive cells was significantly increased after exposure to all Ag- and AuNPs, ranging from an increase with 62–80% compared to control (Figs. 7G–R).

In the INL, no significant increase in TUNEL-positive cells was found after exposure to the four NPs (Figs. 7G–R and B). However, a small increase in numbers of TUNEL-positive cells was found after exposure to 80 nm AgNPs (∼20%) and 20 nm AuNPs (∼10%).

In the GCL, exposure to 20 as well as 80 nm AuNPs significantly increased the number of TUNEL-+ cells in the GCL (62% and 79% compared to control) (Figs. 7G–R and B). In addition, exposure to 80 nm AgNPs increased the numbers of TUNEL-positive cells by 30%, whereas no effect was detected with 20 nm AgNPs.

### Ag- and AuNP exposure cause oxidative stress

**Figure 8:**
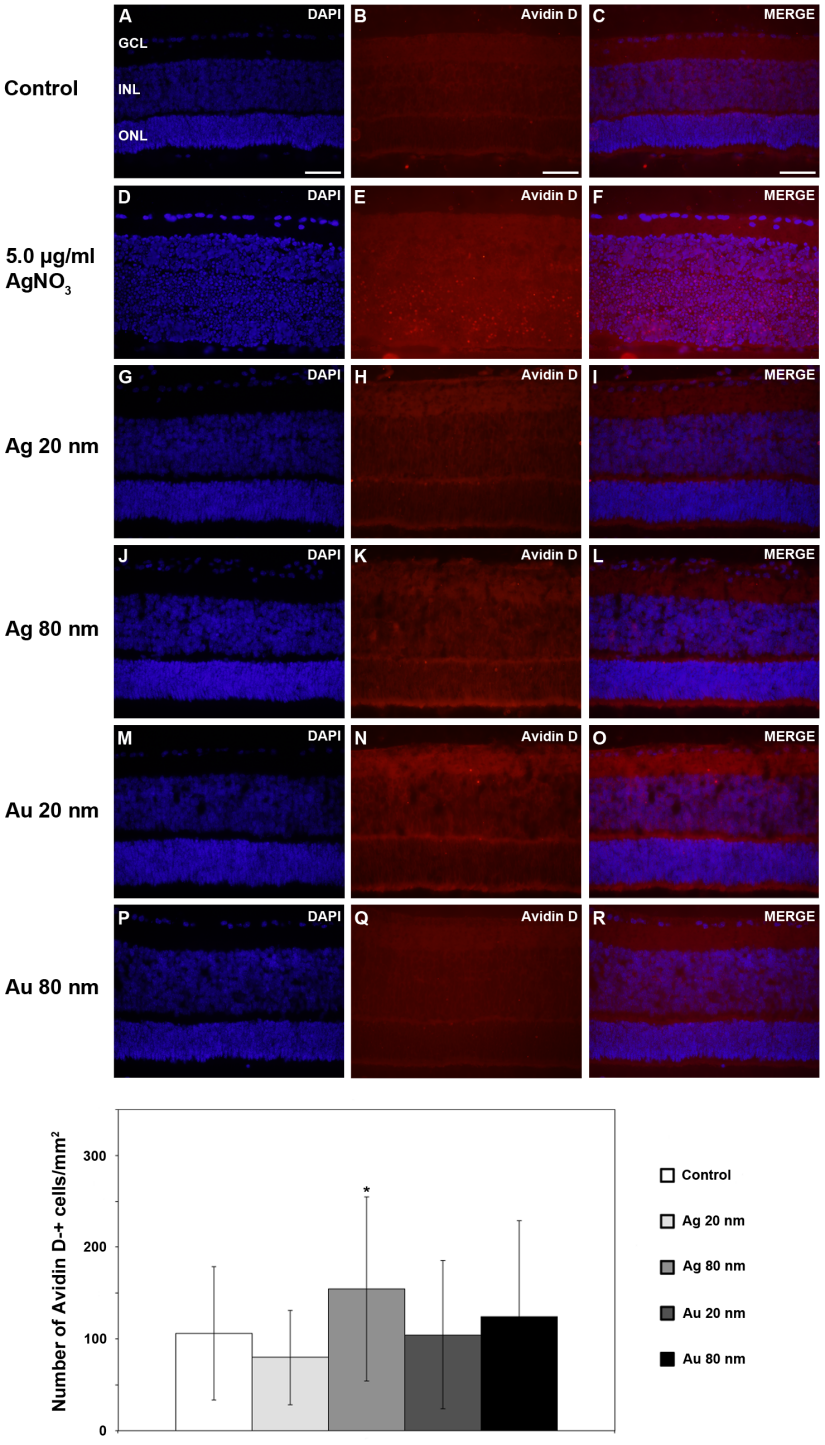
Oxidative stress. Oxidative stressed cells were detected using AvidinD (red) as a marker (DAPI, blue). In the control group (A–C) as well as in the NP-exposed groups the vast majority of AvidinD-positive cells were located within the ONL. AgNO_3_- treated groups displayed abundant numbers of Avidin-positive cells. Graph shows numbers of AvidinD-positive cells and results are presented as mean ±SD (n = 4 explants/group). * *p*<0.05 compared to control. GCL, ganglion cell layer; INL, inner nuclear layer; ONL, outer nuclear layer. Scale bars equal 100 µm.

Avidin can be used to identify oxidatively damaged DNA and has been successfully used in studies of retinal degeneration, since the structure of 8-oxoguanine is similar to that of biotin, *i.e.* the conventional ligand for avidin [27], [28]. In all experimental groups the vast majority of the AvidinD-positive cells found were located in the ONL, with only scattered or no AvidinD-positive cells found in the INL and GCL (n = 4 explants/group). Therefore, the examination of numbers of AvidinD-positive was only performed for the ONL. The significant large number of AvidinD-expressing cells, primarily found in the outer retina confirmed AgNO_3_-toxicity by free Ag ions (Figs. 8D–F).

No difference in AvidinD- positive cell numbers was found between the controls and retinas exposed to 20 nm Ag- and AuNPs (Figs. 8G–I, M–O). In contrast, exposure to 80 nm AgNPs rendered a significant and 47% increase in number of AvidinD- expressing cells (Figs. 8J–L) and exposure to 80 nm AuNPs a non-significant, but 18% increase in numbers of oxidative stressed cells (Figs. 8P–R).

## Discussion

### Low concentrations of Ag- and AuNPs cause unwanted cytotoxic effects

The major findings here were uptake and internalization of 20 and 80 nm sized Ag- and AuNPs into retinal cells, and signs of toxic effects on the retina demonstrated by all parameters studied, *i.e.* gross morphology, glial- and microglial activity and direct cytotoxic effect, including apoptosis and oxidative stress. The selected concentrations for the Au- and AgNPs are based on the low concentrations (0.1–3%) reported to reach secondary organs (including the eye) after systemic administration, and our previous report on uptake and cytotoxicity caused by low concentrations (range 0.002–0.4 µg/ml) using a human neural cell line as model CNS cells [17], [29], [30]. The highest concentration used here is 10 times lower than the concentration used (1.3 µg/ml) of intravitreally injected titanium oxide NP in a very recent study in mouse [31]. Therefore, the concentration of NPs used is relevant with regard to cytoxicity testing of NPs.

Unwanted non-target effects of different NPs, on both primary organs directly exposed, but also to secondary organs, such as the cardiovascular system and the central nervous system (CNS), including the eye have been reported [32], [33]. Neuronal systems (including the retina) are particularly vulnerable, both during development and in adulthood, to metal intoxication. This has also been associated with the development of the major neurodegenerative diseases, such as Alzheimer’s- and Parkinson’s diseases [34], [35].

Today AgNPs and AuNPs are widely used, *e.g.* antimicrobial AgNPs in consumer products and wound dressings, and AuNPs in *e.g.* cancer diagnostics and as carriers for anti-cancer drugs. However, the literature on the toxic side effects on neural tissue by Ag- and AuNPs is very sparse considering it’s wide use in both commercial products as well as in clinical care and diagnostics. Experimental studies show that AuNPs and AgNPs pass the blood-retinal-barrier and are detected in the CNS after systemic administration [36]–[38], *i.e.* AuNPs after intravenous injection in mice [39], [40] and AgNPs after inhalation and subsequently uptake in the respiratory tract [41], [42].

### Models for evaluating the effect of NPs on neural system- use of organotypic cultured post-natal retina

Here we propose a simple assay for initial testing of the effects of metallic- and other NPs on the mammalian retina, using an *in vitro* model of post-natal cultured mouse retina with a defined serum-free culture media [16]. This protocol can easily be modified regarding media composition, NPs, survival times and assays used in order to better mimic certain *in vivo* scenarios. The *invitro* culture system combines accessibility to controlled experimental manipulation, with adherence of whole-mount cultures to a substrate with an excellent preservation of all retinal cell types, numbers and morphology. Over many decades, such culture systems have been used routinely for understanding retinal development, disease mechanisms using *e.g.* genetic models and for examining the potential of innovative therapies [43]–[45]. Notably, with the long-term maintained complex architecture of the retina in culture the present model serves as an excellent model for a neural system as such, including similarity to other neural systems in the CNS. At day 9, *i.e.* the day of NP exposure, the vast majority of the neurons in the explanted retina is post-mitotic and closely resembles the neuronal assembly present in the adult retina [46], [47]. Furthermore, the cultured post-natal rodent retina has proven very useful for studying microglia activation, with the classical hallmarks of inflammation maintained, including morphological changes, cytokine release and proliferation [23]. By post-natal week one-two the entire rodent retina is homogenously populated by microglial cells, making experimentally induced changes easily detectable.

Up-to-date, most studies on neurotoxicity caused by NPs have used *in vitro* systems, including PC-12 cells (a rat cell line with a neuronal-like phenotype) [48], [49], human neural cells [17] and primary neural cultures from mice [50]–[52]. Also, for testing nano-toxic effects in ocular research, the majority of reports are based on *in vitro* studies, including retinal pigment epithelium (RPE) cells [53]–[55]. However, there are a few *in vivo* studies reporting assessment of nanoparticle-related toxicity to the eye and the retina [4], [6], [11], [12], [56], [57]. Notably, recent used cell assays for testing NP-related toxic effects rarely include neuronal cell-based assays, the most vulnerable cell type in the retina. Taken together, the present used organotypic retinal explant model indeed serve as a useful medium-throughput screening tool for investigations of the potential use of various NPs in a neural system, especially the retina.

### Internalization of Ag-and AuNPs within the nucleus and cytoplasm of retinal cells

TEM analysis clearly revealed uptake of all studied NPs into every neuronal layer of the retina, with the main difference that 20 nm NPs were more frequently taken up into the nucleus compared to the 80 nm NPs. After 72 h of exposure all four types of NPs could be found as single NPs or compact NP-aggregates in the nucleus or cytoplasm of the cells in the three neuronal layers of the retina. Today there are no reports describing whether NPs are taken up individually or as aggregates over the cell membrane. It can be speculated that smaller NPs are taken up only when a critical density is reached locally, and are thus taken up as aggregates [58]. Since only one time-point was studied here it was not possible to examine whether a larger fraction of NPs was present as individual NPs early after administration.

Up-to-date the vast majority of reports on cellular uptake of Ag- and AuNPs only describe these NPs localized to the cytoplasm [59]–[62]. The reports showing internalization of 20–80 nm sized Ag- and AuNPs in the nucleus of eukaryotic and more specifically mammalian cells are very sparse. However, Hackenberg *et al* showed uptake of AgNPs (<50 nm) into the nucleus of mesenchymal stem cells [60], and we reported, uptake of 20 and 80 nm Ag- and AuNPs by human neural cells as well as into the nucleus using two different models for studying uptake, *i.e.* expansion- and differentiation conditions [17] (and unpublished data).

It is remarkable that we find both 20 and 80 nm sized Ag- and AuNPs within the nucleus, since the nuclear pore can only be dilated to around 39 nanometers to allow passage of various molecules [63], [64]. Thus, it can be expected that 20 nm NPs are transported through the nuclear pore, but not the 80 nm NPs. The localization of 80 nm sized NPs in the nucleus may be explained by uptake into cells undergoing early apoptosis where the nuclear envelope is perturbed [65]. This is in line with the significant increase in numbers of TUNEL-positive cells found after NP-exposure. In addition, uptake of AuNPs >50 nm have been previously reported (56) which suggests that NPs may interfere with the nuclear pore allowing for uptake.

With regard to the cytotoxic effect, the findings that a larger fraction of the 20 nm sized NPs were detected in the cell nuclei compared to NPs found in the cytoplasm is highly important and interesting. In addition, only the 20 nm sized NPs were found within mitochondria.

### Cellular level effects of Ag- and AuNP exposure in retinal tissue: increased apoptosis oxidative stress and microglia activation

Changes in the end-points included are well described as signs of insult to neural tissue including after NP exposure, *i.e.* gross morphological organization, glial activity, microglial activity, level of apoptosis and oxidative stress.

At the cellular level, cytotoxicity caused by Ag- and AuNPs was assessed studying the numbers of apoptotic- and oxidatively stressed cells, respectively. In the control retina apoptosis was found in all layers, which is expected since the explantation process itself cause a stress response in the tissue [16]. Overall, after exposure to all Ag- and AuNPs, numbers of apoptotic cells, detected by using dUTP TUNEL staining, were significantly increased. This was true especially for the photoreceptor layer located in the outer retina (*i.e.* the ONL), and the ganglion cell layer in the inner retina (GCL), but with no significant effect on numbers of apoptotic cells was found in the mid-nuclear layer (INL). The absence of induction of apoptosis in the INL may be explained by the central location of this layer in the retina and thus a longer distance for the NPs to travel. In agreement, the photoreceptors are described to be very vulnerable to environmental insult, such as foreign particles or light damage compared to the other retinal neurons [66]. In the photoreceptor layer no difference was seen in toxicity caused by Ag- compared to AuNPs of the same sizes. However, in the GCL a tendency toward more toxicity caused by the AuNPs of both sizes compared to their Ag counterpart NPs was noted.

Our results are overall in agreement with several recent reports using different assays describing interference with DNA regulation after Ag- and AuNP exposure [67]. In mouse embryonic stem cells, AgNPs induce apoptosis shown by increased Annexin V expression, up-regulation of p53, and DNA damage [34]. AgNPs were shown to induce cell death in a size and dose dependent manner in a primary mixed neural cell culture, while AuNPs had no effect, using a lactate dehydrogenase (LDH) assay [68]. The activation of the transcription factor NFκB, an immediate early response gene to injury, is another way to study apoptosis, and large AuNPs have been found to induce activation of the NFκB pathway due to the oxidative stress, while small AuNPs have no effect [62], [67].

Evaluation of induction of oxidative stress by the NPs revealed only AvidinD- positive cells in the ONL and none found in the GCL and INL, which is in agreement with the photoreceptors being the most vulnerable retinal neurons. Oxidative stress at high levels is known to lead to cell injury and death and several reports on nanotoxicity caused by Ag- and AuNPs mainly from cell-based assays describes induced oxidative stress. AuNPs induce oxidative stress in neural progenitor cells [62] and AgNPs induce oxidative stress in skin fibroblasts and airway smooth muscle cells [69], [70]. Lately, oxidative stress responses including interference with calcium-based signaling processes were reported for AgNPs, using mixed primary neural cultures [68]. Other types of NPs also cause oxidative stress, *e.g.* in endothelial and epithelial cells, measured by an increase in ROS or in nitric oxide production two messengers known to modulate *e.g.* inflammatory reactions [71].

Our contradictory results to others results on especially AuNP-related toxicity, with no or minimal toxic effect to retinal cells, may be explained by the different assay, NP size, exposure time and analysis method used. In *in vivo* studies, the milieu affecting the injected NP are much more complex than in our *in vitro* system, where both the retinal blood supply and RPE cells are missing [33], [40]. It may be suggested that the NP-related neuronal toxicity in *in vivo* studies is milder since *e.g.* the surface chemistry of the NP is changed and the immune response is protecting the retinal neurons upon NP exposure. In accordance, intravenously administered AuNPs induce no retinal toxicity as reported by Kim *et al*, 2009 [40]. However, compared to our results Kim *et al* (2009) studied the retina 24 h after administration and their TEM analysis localized NPs only in the cell membranes. Olson *et al* (2013) report maintained function up to 6 weeks after intravitreal injection of 5 nm AuNPs. In the same report a decrease in viability is demonstrated using a RPE cell line. RPE cells, together with retinal astrocytes and retinoblastoma cells are examples of cell-based assays, *i.e.* non-neuronal, utilized to study retinal toxicity of AuNPs [33], [72]. Prolonged microglial activation is associated with retinal degeneration and photoreceptor apoptosis. Numbers of microglial cells were increased 12–53% after exposure to all four NPs studied. Proliferation of microglial cells is one mechanism induced upon inflammatory response. However, the only significant increase in numbers was found after exposure to the 80 nm AuNPs. In parallel, 80 nm AgNPs were the only NPs significantly showing higher numbers (30%) of activated microglial profiles after exposure. In addition, exposure to 20 and 80 nm AgNPs rendered a 15 and 11% increase of activated microglial morphologies, although not significant.

The markers used here, Iba1 and ED1 for evaluating microglial numbers and level of activation based on morphological characterization of the cells is well-described within research on neuroinflammation [73].

Recently, four inorganic NPs in the size range 20–60 nm were reported to induce microglial activation using a microglia cell line, that further by production of pro-inflammatory cytokines induced cytotoxicity and dysfunction in a neuronal-like cell line (PC12 cells) [74]. In addition, abundant experimental evidence has demonstrated that inflammation mediated by microglia contributes to neurodegenerative diseases, including Parkinson’s disease and Alzheimer’s disease [75]–[77].

### Tissue level effects of Ag- and AuNP exposure in retinal tissue- gross morphological changes and glial activation

The retina contains a diverse range of cell types, and these are arranged in a very precise spatial organization, required to achieve detailed vision. If the linear structure of the retina is distorted by an insult there is a great risk of an interrupted visual pathway, and subsequently visual impairment or loss.

A similar stress response was found for all Ag- and AuNPs studied at a gross morphological level demonstrated by the presence of vacuoles typically within the INL and an overall impression of more densely labeled and packed cells in the ONL. In addition, the thickness of the retina from one eccentricity to the other tended to be larger compared to control. This is in agreement, with our recently reported NP-related effects on gross morphology of human neural cells using the same NPs [17]. This point is of particular interest since the different components of the cytoskeleton interact to control multiple functions, including migration, cell morphology, structural integrity, division, deformation, intracellular transport and tissue organization. Furthermore, others and we have recently described changes in the expression of the microfilament actin using cell-based assays after Ag- and AuNP-exposure [17], [62].

Changes in intermediate filament GFAP staining pattern and expression level were clear for both 20 and 80 nm NP exposure. GFAP up-regulation is well described to be a hallmark for retinal injury, and is associated with normal Müller cell regulation of retinal homeostasis. Retinal neurons are dependent on the Müller cells to maintain their cellular microenvironment and stability of the entire retinal sheet. Exposure to the 80 nm sized NPs rendered a more pronounced activation of GFAP compared to their smaller 20 nm-sized counterparts. For both 20 and 80 nm NPs, up-regulation of GFAP expression was detected by a larger fraction of GFAP-positive thin and thick processes found throughout the retina compared to control. In addition, only in the 80 nm NP exposed retinas GFAP-positive processes with irregular shapes were found. Also in our previous study, the 80 nm sized NPs activated GFAP the most [17]. Taken together, the activation of glia and also change in morphologies of the processes of glial the Müller cells, with the important function to maintain the retinal structure, may indicate that there is a risk for neuronal dysfunction of the retina and thus a risk of visual impairment.

## Conclusions

The main findings of the present study were that low concentrations of 20 and 80 nm sized Ag- and AuNPs have unwanted effects on the retina. All tests included here revealed the NPs impact on cellular and tissue levels, *e.g.* a significant neurotoxic effect especially on the sensory neurons of the retina, the photoreceptors. The photoreceptors are the most vulnerable neurons of the retina and the key to proper vision – insult of these cells therefore lead to visual impairment or even blindness. Therefore, all NP that may reach the eye, especially the retina, in ocular therapies or accidently from *e.g.* cosmetics and environmental pollution should be carefully evaluated in relevant models of the eye and the retina. The *in vitro* retina tissue model presented here is excellent for initial testing of neurotoxicity of candidate NPs, since it offers a maintained long-term retinal organization and controlled serum-free conditions.
